# Continental Shelf Sediments of Sarawak, Malaysian Borneo

**DOI:** 10.1155/2017/4853048

**Published:** 2017-09-18

**Authors:** Wan Zabidii Wan Morni, Siti Akmar Khadijah Ab Rahim, Tarmiji Masron, Richard Rumpet, Jamil Musel, Ruhana Hassan

**Affiliations:** ^1^Faculty of Resource Science and Technology, Universiti Malaysia Sarawak, 94300 Kota Samarahan, Sarawak, Malaysia; ^2^Faculty of Social Science, Universiti Malaysia Sarawak, 94300 Kota Samarahan, Sarawak, Malaysia; ^3^Fisheries Research Institute Sarawak, Department of Fisheries Malaysia, P.O. Box 2243, 93744 Kuching, Sarawak, Malaysia

## Abstract

Sediment distributions in deep sea influence the benthic community structure and thus play an important role in shaping the marine ecosystem. Several studies on sediment characteristics had been conducted in South China Sea (SCS), but only limited to coastal areas of regions within SCS territories. Therefore, this study was carried out to analyze the benthic sediment profile in an area beyond 12 nautical miles off the coast of Sarawak, southern SCS. Sediment samples were collected from 31 stations, comprising three depth ranges: (I) 20–50 m, (II) 50–100 m, and (III) 100–200 m. The total organic matter (TOM) contents were determined and subjected to dry and wet sieving methods for particle size analysis. TOM contents in the deep area (>50 m) were significantly higher (*p* = 0.05) and positively correlated (*r* = 0.73) with silt-clay fraction. About 55% and 82% of stations in strata II and III, respectively, were dominated by silt-clay fractions (<63 *μ*m mean diameter), coherent with TOM data. In addition, sediments in the deep area (>50 m) tend to be poorly sorted, very fine skewed, and platykurtic. Unlike data obtained 20 years ago which reported high content of silt-clay (58%), this study recorded a lower content (35%); therefore, changes in sediment load had been observed in southern SCS.

## 1. Introduction

The South China Sea (SCS) is part of the Pacific Ocean, encompassing an area from Karimata and Malacca Straits to the Taiwan Straits with approximately an area of 3,500,000 km^2^ [1]. Sediment contents in the SCS are generally volcanic, biogenic, and terrigenous material [2], which also consists of illite, chlorite, kaolinite, smectite, and mixed-layer clay minerals [3]. Sediment plays an important role in structuring deep sea communities because deposit feeders rely on nutrition from sediments and comprise most of the marine organisms in the sea [4]. The habitat complexity was reflected by particle size diversity where the organisms live on or within the sediments [5]. The nature of sediment is based on the complex interaction of various factors that determine sediment material, transportation, deposition, and postdeposition of sediment. Moreover, it all depends on the source of material, either geological or biological in origin or both, in order to determine the composition [6]. The analysis of particle size therefore provides important clues on the sediment origin, depositional condition, and transport history [7].

It is presumable that the sedimentary environment will continue unchanged if the interaction remains stable unless some changes take place in any of the factors and within a period of time, which led to the alteration of sediment [8]. Sediment transport is one of the factors that are much more influenced by water current and the mobility of particles which depends on roughness velocity, threshold velocity, settling velocity, and water velocity [9]. Coarse sediment such as pebbles and cobbles from the beach origin can be transported to the deep sea area by the force of waves [10]. Basically, coarse particles are found on the bottom of the fast flowing area, while fine sediments are deposited at deep and quiet waters in the offshore areas [11].

According to Kao et al. [12], more than 80% of the average of organic matter buried on the continental shelf derived from rapid sedimentary accumulation and high biological production. Hence, the large proportions of the nutrients derived from the decomposition of organic matter on the bottom will determine the productivity of the primary producer (phytoplankton) [13]. Additionally, the continental shelf is the underwater extension of the continent which gradually slopes from shore to depth of 200 m [14]. Within this area, the organic matter from inshore is mainly derived from rivers; however, beyond that, it is mainly derived from marine planktonic algae [15].

Previously, there were a large number of literatures on sediment profile in the continental shelf of Sarawak. For example, Chong and Idrus [16] focused on zinc and copper distribution associated with sediment structure, while, in 1996, Husain et al. [17] and Shazili et al. [18] conducted studies on sediment characteristics and trace metals, respectively. However, information on benthic sediment profile with depth influenced is still limited and not much discussed. Recently, the National Demersal Fish Resource Survey was conducted in the Sarawak Exclusive Economic Zone (EEZ) area whereas a surface sediment sample was collected. This study was carried out to analyze the benthic sediment profile according to depth in the southeastern part of SCS.

## 2. Materials and Methods

### 2.1. Sediment Collections

**Figure 1:**
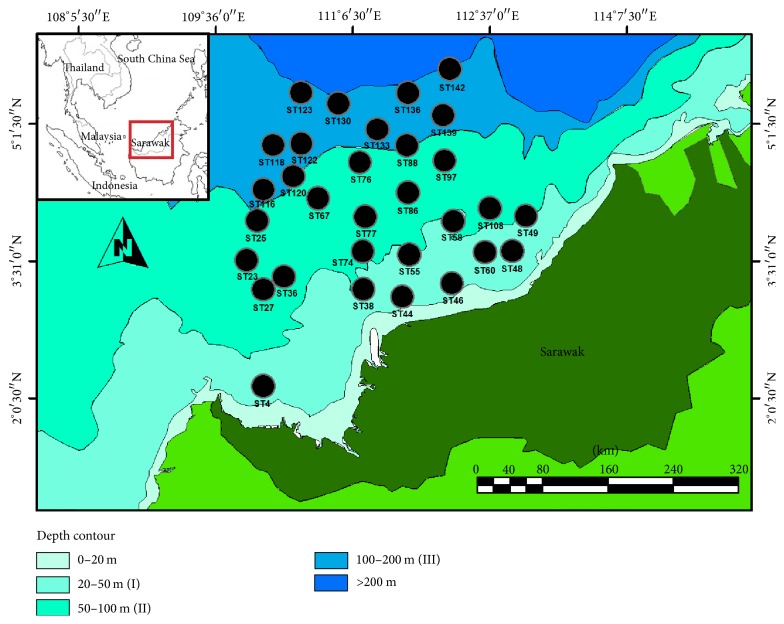
Thirty-one (31) sediment sampling stations in the continental shelf of Sarawak.

A three-month (16 August until 6 November 2015) sampling cruise was carried out in an area beyond 12 nautical miles off the coast of Sarawak. Sediment collection is one of the side activities from the main survey (demersal fish resource assessments) of the cruise. The study area comprised sandy, muddy, and rocky bottoms and hard corals, besides consisting of sloping areas with a depth of more than 200 meters. Samples were collected from 31 selected stations (Figure 1) which comprised three depth ranges following Morni et al. [20]: (I) 20–50 m, (II) 50–100 m, and (III) 100–200 m. The research vessel MV SEAFDEC 2 owned by Southeast Asian Fisheries Development Centre (SEAFDEC) was used throughout the survey. Surface sediment samples for total organic matter (TOM) and particle size analysis (PSA) were collected using Smith McIntyre grab sampler. Three replicates of samples were taken and placed inside the labeled plastic bag before being kept at −20°C during the field survey before being transported to the laboratory for analysis.

### 2.2. Sediment Analysis

#### 2.2.1. Total Organic Matter (TOM)

The total organic matter (TOM) analysis was conducted to examine the loss of weight on ignition and a method by Greiser and Faubel [21] was used to determine the amount of TOM in sediments. This method involves drying of wet sediment (25 g) at 60°C for 24 h to determine the initial dry weight value. The dried samples then were combusted at a high temperature of 475°C for 2 h to determine the final weight. The equation involved initial and final weight value to determine the weight loss:

(1) $$\begin{array}{c} \%\;\;TOM=\frac{\left(A-B\right)}{A}*100, \end{array}$$

where *A* is the initial dry weight (60°C, 24 hours) and *B* is the final weight (475°C, 2 hours).

#### 2.2.2. Grain Size Analysis

Grain size analysis defines the sedimentary environment characteristic with a series of particle size distributions. The analysis was conducted to characterize the continuous distribution from small particles to many millimeters of sediment fractions. Dry sieving and wet sieving methods were used in the measurement of sand and silt-clay fraction, respectively, following the method by Holme and McIntyre [8]. The method involves a splitting process of sediment (30 g) into different grades of sand (very coarse sand: 1000 *μ*m; coarse sand: 500 *μ*m; medium sand: 250 *μ*m; fine sand: 125 *μ*m; very fine sand: 63 *μ*m) and silt-clay fraction (particle size <63 *μ*m). Udden-Wentworth's [22, 23] grade scale was used to determine fraction characteristics.

### 2.3. Statistical Analysis

The 3D graph of TOM by depth strata was plotted using OriginLab 9.0™ software. All data were subjected to one-way analysis of variance (ANOVA) and multiple-comparisons (Tukey's) test was used to examine significant differences among averages of mean value of stations in all contours. Correlation analysis was used to compare differences of mean between TOM with sediment fractions with significance level at 0.05 using Minitab 17™.

In order to compare sedimentary environments with each other quantitatively, four parameters were used to describe the grain size distribution: (a) the particle average size, (b) the spread (sorting) of the sizes around the average, (c) the symmetry or preferential spread (skewness) to one side of the average, and (d) the degree of concentration of the grains relative to the average (kurtosis). These properties were determined by the logarithmic method of moments and descriptive terminology (Table 1) by Krumbein and Pettijohn [19].

### 2.4. Visualization and Mapping

Mapping technique to portray information into a map is the common and basic study on marine geographical information systems (GIS) works. Distributions mapped in this way provide a good visual relationship between sediment characteristics with sea depth and distance to coastline. Sediment data obtained from Sarawak waters were presented into a map using ArcGIS 10.1™ software. The information of total organic matter (TOM), sea depth, and sampling location (latitude and longitude) was compiled into visualized information into maps.

## 3. Results

### 3.1. Total Organic Matter (TOM) of Sarawak EEZ

**Figure 2:**
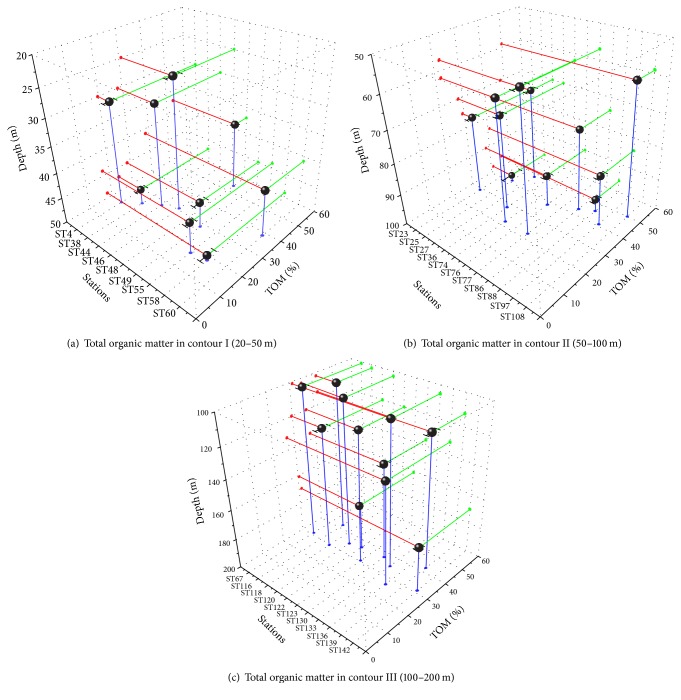


As can be seen from Figure 2(a), the lowest amount of TOM in contour I was recorded in station 55 (17.30 ± 1.97%) at a depth of 44 m, while the highest TOM in contour I was recorded from station 48 (53.50 ± 1.23%) at a depth of 37 m. In contour II (Figure 2(b)), the lowest amount of TOM was recorded in station 74 (22.16 ± 0.38%) at a depth of 62 m, while the highest TOM in contour II was recorded at station 108 (50.12 ± 1.00%) at a depth of 59 m. Meanwhile, in contour III (Figure 2(c)), the lowest amount of TOM value was recorded in station 136 (26.62 ± 0.94%) at a depth of 132 m and the highest TOM value recorded was in station 139 (42.41 ± 2.84%) at a depth of 112 m. Therefore, the averages of the mean values of TOM in contours I, II, and III were 28.66 ± 11.50^a^%, 37.19 ± 10.08^b^%, and 34.31 ± 5.08^ab^%, respectively. These results had shown that contour II had significantly higher organic matter compared to contour I whereas there is no difference of TOM value with contour III.

**Figure 3:**
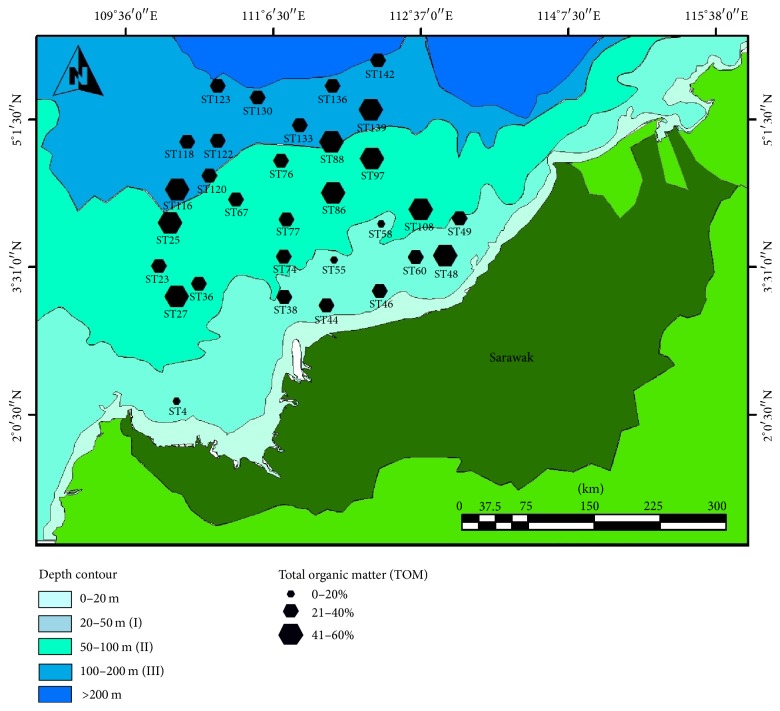
Amount (%) of TOM at 31 stations in the continental shelf of Sarawak.

Figure 3 summarized the average values of TOM in 31 sampling stations, with three different sizes of hexagons which indicated the amount of TOM that ranged from 0 to 20% (3 stations), 21 to 40% (19 stations), and 41 to 60% (9 stations), respectively.

### 3.2. Particle Size Distribution in Sarawak EEZ

**Figure 4:**
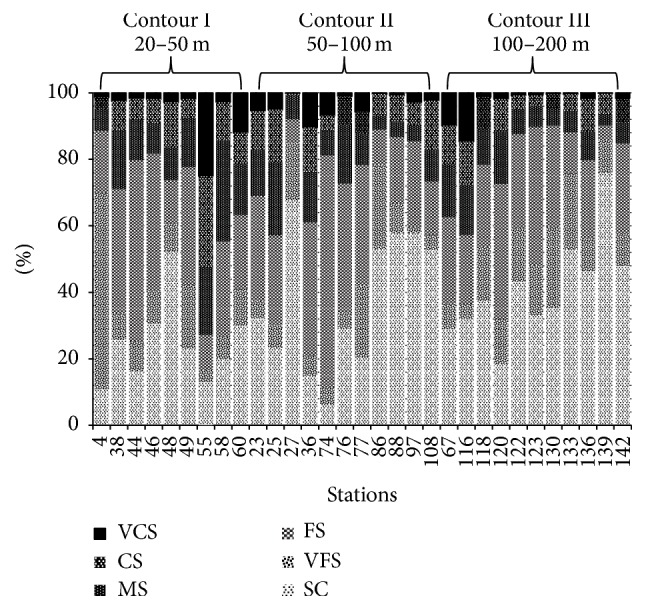
The particle size distribution of 31 stations sampled at Sarawak EEZ. VCS: very coarse sand (1000 *μ*m); CS: coarse sand (500 *μ*m); MS: medium sand (250 *μ*m); FS: fine sand (125 *μ*m); VFS: very fine sand (63 *μ*m); SC: silt-clay (<63 *μ*m).

In the present study, the distributions of particle size vary according to station (Figure 4). Sediment fractions with sizes of 1000 *μ*m, 500 *μ*m, 250 *μ*m, 125 *μ*m, 63 *μ*m, and <63 *μ*m are referred to as very coarse sand (VCS), coarse sand (CS), medium sand (MS), fine sand (FS), very fine sand (VFS), and silt-clay (SC), respectively. Out of 9 stations in contour I, 4 stations recorded the highest percentage of FS fractions. Meanwhile, in contour II, out of 11 stations, 6 stations recorded the highest percentage of SC fractions. For contour III, out of 11 stations, 9 stations recorded a high percentage of SC fraction.

**Figure 5:**
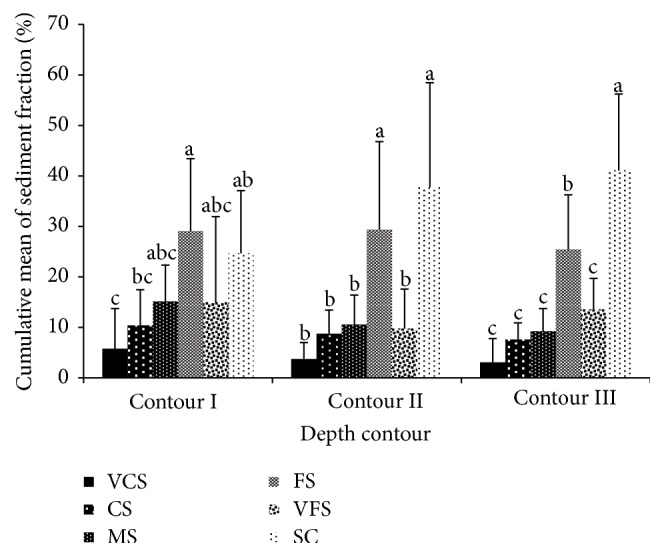
The average means of sediment distribution at contours I, II, and III. VCS: very coarse sand (1000 *μ*m); CS: coarse sand (500 *μ*m); MS: medium sand (250 *μ*m); FS: fine sand (125 *μ*m); VFS: very fine sand (63 *μ*m); SC: silt-clay (<63 *μ*m). Means that do not share a letter are significantly different.

These results had shown that FS was dominantly found in contour I, while SC was dominantly found in contour II and contour III, respectively. Thus, the average means were calculated to summarize sediment distributions of each depth contour.  Figure 5 shows that the highest sediment fractions in contours I, II, and III are FS, SC, and SC, respectively; meanwhile, the VCS fractions were recorded as the lowest grain size fractions in all depth contours.

**Figure 6:**
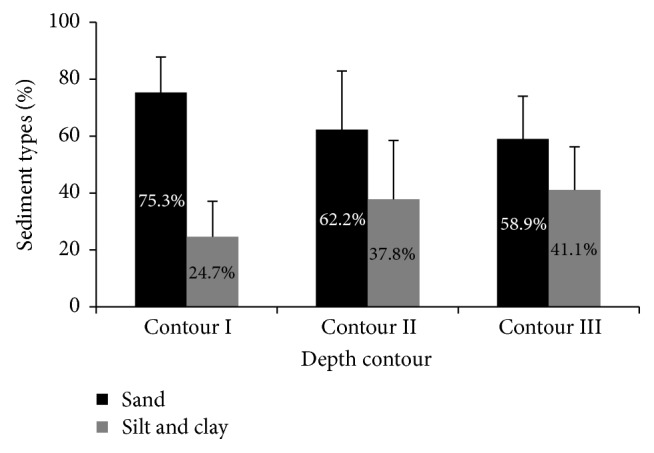
The average percentage of sand and silt/clay of all depth contours.

Figure 6 summarizes the average percentage of sand and silt-clay in every contour. Averages of sand content in contours I, II, and III are 75.3%, 62.2%, and 58.9%, respectively. Meanwhile, the average percentages of silt-clay in contours I, II, and III are 24.7%, 37.8%, and 41.1%, respectively. From coastal to deeper areas, sand contents in sediment had shown a decreasing trend, while silt-clay content gradually increased.

**Figure 7:**
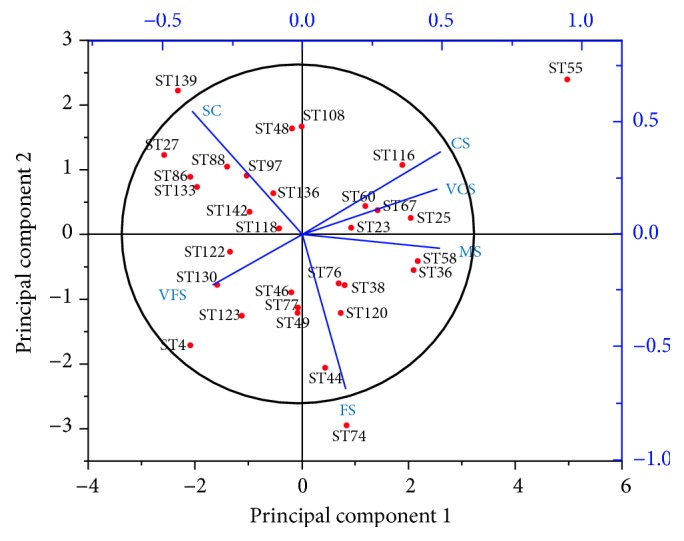
The principal component analysis (PCA) ordination derived from the percentage of grain size analysis in all stations. VCS: very coarse sand (1000 *μ*m); CS: coarse sand (500 *μ*m); MS: medium sand (250 *μ*m); FS: fine sand (125 *μ*m); VFS: very fine sand (63 *μ*m); SC: silt-clay (<63 *μ*m).

Figure 7 illustrates the results of principal component analysis (PCA) of mean percentage of sediment at all stations. The highest sediment fraction in the continental shelf of Sarawak is SC (35.15%), followed by FS (27.88%), VFS (12.62%), MS (11.43%), CS (8.82), and VCS (4.10%). Apart from that, only stations 55, 74, and 139 were found as outliers due to the high percent of CS, FS, and SC, respectively, which exceeded the range of values recorded by other stations.

### 3.3. Mean, Sorting, Skewness, and Kurtosis Coefficient Value

Statistical analysis was adapted to the sediment size frequency and sediment was described in terms of analysis of mean size, sorting, skewness, and kurtosis (Table 2). The average of the mean size was higher in contour I (1.58Φ), followed by contours II (1.34Φ) and III (1.33Φ). Therefore, measuring the scatter of the central value (sorting) was conducted. In contours I, II, and III, sorting coefficient was in the range of 0.72 to 1.12Φ which is considered moderately sorted to poorly sorted and classified as FS. Out of 9 stations, there are 7 and 2 stations in contour I that showed moderate sorting and poor sorting of particle distribution in sediment, respectively, while in contour II, out of 11, there are 6 and 5 stations that showed moderate sorting and poor sorting of particle distributions, respectively. In contour III, out of 11, there are 5 and 6 stations that demonstrated moderate sorting and poor sorting of particle distribution, respectively.

The degree of asymmetry (skewness) of the sediment in Sarawak EEZ was dominated by fine sediment which is represented by a positive (+) value. Therefore, in contour I, there are 1, 7, and 1 stations that showed very fine skewed, fine skewed, and coarse skewed sediment distribution frequency, respectively. In contour II, there are 5, 3, 2, and 1 stations that showed very fine skewed, fine skewed, symmetrical, and very coarse skewed sediment distribution frequency, respectively. In contour III, there are 7, 3, and 1 stations that showed very fine skewed, fine skewed, and symmetrical sediment distribution frequency, respectively.

The degree of peakedness (kurtosis) of the grains relative to the average was calculated. In contour I, 56%, 22%, and 22% of the stations showed platykurtic, mesokurtic, and leptokurtic characteristics, respectively. For contour II, 45%, 27%, and 27% of the stations showed platykurtic, mesokurtic, and leptokurtic characteristics, respectively. In contour III, 72%, 18%, and 9% of the stations showed platykurtic, mesokurtic, and leptokurtic characteristics, respectively. Lastly, as a summary, from all 31 stations sampled, 58%, 23%, and 19% of the stations showed platykurtic, mesokurtic, and leptokurtic characteristics, respectively.

## 4. Discussion

The fine fraction sediment forms a good bonding with the organic compound through strong cation exchange capacities for organic adsorption on the sediment surface [24, 25]. According to Rashid [26], the sediment particle size plays a role in bonding the organic compound whereas 10 to 20% of the organic matter is believed to bond with fine particles. Apart from that, most organic contents are processed by heterotrophic microorganisms that use organic compounds such as carbon as nutrient and energy source [27]. Therefore, the growth rate of microbes is controlled by the availability of nutrients which can be found in the organic matter and soil solution.

Basically, it was suggested that the organic compounds are well composed in fine or silt-clay fraction. Similarly, in the present study, positive correlation was obtained between organic matter and silt-clay fraction (*r* = 0.73), resulting in a high proportion of organic matter in sediments of the continental shelf of Sarawak. For example, in contour I, the highest silt-clay fractions were at station 48 which also recorded high organic content (53.5%). Apart from that, the high content of sand fractions showed a low amount of total organic content. As an example in contour I, the highest sand fractions were at station 74 which recorded a low amount of organic matter (22.2%). Thus, the results obtained showed a similar trend to studies that were conducted in the continental shelf of Southern California [28], Vietnam [29], Northwest Peninsular Malaysia [30], Northern Spain [31], and South Yellow Sea of China [15]. According to Kao et al. [12], more than 80% of organic matter was buried in the continental shelf. These euphotic zone areas comprise light and nutrients that enhance plankton production which contributes to the accumulation of organic matter in the life cycle of planktons [13, 14]. The dead and decaying planktons constitute the richest sources of amino acids, carbohydrates, fatty acids, lipids, proteins, and other organic matter compounds [32].

Based on the average of particle size analysis from all stations, the current study demonstrated a higher percentage (65%) of sand than the data obtained from the last two decades of survey whereas only 42% of sand had been reported by Husain et al. [17]. However, the percentage of silt-clay fraction in the present study (35%) was lower than the percentage reported by Husain (58%). Surprisingly, the amount of sand on the surface sediment of Sarawak waters is likely increased, while the silt-clay proportion decreased. This situation is closely related to the sediment supply and the process of transportation of sediment from the coast to the ocean [33, 34] and is associated with the current pattern. Over time, the gradual rising of sea level and weathering process could lead to increasing the shoreline erosion, sedimentation, and sediment accumulation process [35]. In addition, the sediment alteration can be influenced by anthropogenic factors such as bottom trawling activity which could induce sediment flux 6 times greater than water current and wave sediment flux [36].

A previous study conducted by Gorsline [37] showed that most of the mean diameters of surface sediment particles in the continental shelf of South Atlantic, United States, ranged from 250 to 500 *μ*m. In contrast, during this study, in contour I, 44% of the mean diameters ranged from 125 to 250 *μ*m. Meanwhile, in contours II (55%) and III (82%), majority of the mean diameters of surface sediment are less than 63 *μ*m. In addition, Figure 7 shows that the FS, VFS, and SC structures were the most discriminant size fractions. This can be affected by monsoon factors where the present study was conducted in premonsoon seasons. This is presumably caused by low energy factors (bottom current activity and wave) in the continental shelf of Sarawak which is responsible for the high accumulation of fine particle sediments. The sizes of particles were also significantly affected by the northeast and southwest monsoon in southern SCS [38]. For example, Husain et al. [17] and Kamaruzzaman et al. [39] reported that the sizes of particles were finer during postmonsoon period compared to the premonsoon season in southern SCS. According to Gray [9], the most stable size of particle diameter is 180 *μ*m and this value is within medium to fine sand fraction where it is the best sorting value as reported by Inman and Chamberlain [34]. A particle of this size moves easily compared to the coarser particle that is much denser, which restricts transportation. Sediments are considered stable at near-shore areas where less movement occurred compared to the deeper areas that were influenced by strong water currents [9]. This phenomenon indirectly influences the size of particles that were deposited on the sea bottom. According to Shepard et al. [40], environmental factors that were responsible for irregular distributions of grain size are water currents, large wave exposure, proximity to large river mouth, adjacency to sandy beaches, and abundance of calcareous organisms.

In the present study, 78%, 54%, and 45% of stations in contours I, II, and III, respectively, demonstrated sediments that were moderately sorted. Meanwhile, 22%, 45%, and 54% of stations in contours I, II, and III, respectively, demonstrated sediments that were poorly sorted (Table 2). Similarly, studies done by Husain et al. [17] and Gorsline [37] showed that the deeper sediments tend to be more poorly sorted compared to the shallow area. The poorly sorted sediment is heterogeneous and synonymous with low wave and current activity, while well sorted sediment is homogeneous which is typically synonymous with high wave and current activity [9]. Moreover, under the extreme wave conditions, fine sediments resuspended over all of the sea floor area [31].

At a depth of 50–200 m in the present study, a high excess of fine sediment or very fine skewness was recorded. However, the study conducted by Husain et al. [17] reported that the symmetrical excess (medium size particle) of sediment was dominant in midshore and offshore of Sarawak waters during premonsoon seasons in 1996. This could be associated with the fluctuating intensity of sediment texture that was affected mainly by current activity and wave action [11]. In the present study, 19%, 23%, and 58% of the stations showed leptokurtic (excessively peaked), mesokurtic (natural sediment average), and platykurtic (deficiently peaked) curve, respectively. The leptokurtic one can be considered as a better sorted distribution in the central area of the curve, while the mesokurtic one was nearly normal and the platykurtic one is flat peaked [8]. Therefore, the results obtained provide valuable information on alterations that occurred in sediment accumulation status in Sarawak's continental shelf. It is important to measure the rate of changes in sediment characteristics as a preparation to overcome negative implications to the species biodiversity and marine ecosystem overall.

## 5. Conclusion

This study provides useful information on the sediment profile in the continental shelf of Sarawak. High amounts of TOM were positively correlated with silt-clay fractions. For the sediment distribution, FS was dominantly deposited in contour I, while SC was dominantly deposited in contour II and contour III, respectively. The highest grain size distribution in the continental shelf of Sarawak is SC, followed by FS, VFS, MS, CS, and VCS. In addition, deeper sediments tend to be more poorly sorted, very fine skewed, and platykurtic compared to the shallow area. The increment of sand fractions in the surface sediment of the continental shelf of Sarawak should be seen as a serious problem. Therefore, there is a need for further research, in order to investigate the recent status of benthic sediment alteration for better management and actions by relevant agencies.

## Figures and Tables

**Table 1:** Descriptive terminology modified from Krumbein and Pettijohn [19].

Sorting (**σ**Φ)		Skewness (SkΦ)		Kurtosis (KΦ)	
Very well sorted	<0.35	Very fine skewed	>+1.30	Very platykurtic	<1.70
Well sorted	0.35–0.50	Fine skewed	+0.43 to +1.30	Platykurtic	1.70–2.55
Moderately well sorted	0.50–0.70	Symmetrical	−0.43 to +0.43	Mesokurtic	2.55–3.70
Moderately sorted	0.70–1.00	Coarse skewed	−0.43 to −1.30	Leptokurtic	3.70–7.40
Poorly sorted	1.00–2.00	Very coarse skewed	<−1.30	Very leptokurtic	>7.40
Very poorly sorted	2.00–4.00				
Extremely poorly sorted	>4.00				

**Table 2:** Sand and silt/clay fraction distribution calculated using the method of moments statistical analysis.

Strata	Station	Depth (m)	Mean size	Sorting	Skewness	Kurtosis
I	4	30	2.51	0.72	−0.93	4.80
	38	47	1.62	0.89	0.76	2.15
	44	30	2.01	0.76	−0.02	2.61
	46	25	1.63	0.92	0.89	1.98
	48	37	0.88	0.99	1.85	3.94
	49	45	1.86	0.86	0.60	2.19
	55	44	0.88	1.01	0.80	2.59
	58	49	1.59	0.84	0.70	2.47
	60	41	1.26	1.10	0.78	2.12

II	23	75	1.34	0.99	0.94	2.19
	25	98	1.43	0.95	0.75	2.26
	27	70	0.87	1.10	1.95	3.90
	36	70	1.61	1.05	−0.01	1.88
	74	62	2.19	0.87	−1.54	4.85
	76	90	1.57	0.85	1.06	2.13
	77	57	1.86	0.99	0.07	2.20
	86	74	1.21	1.12	1.62	2.77
	88	96	0.96	1.05	1.79	3.38
	97	84	0.88	1.02	1.70	3.24
	108	59	0.84	0.95	1.90	4.06

III	67	101	1.28	1.05	0.78	2.15
	116	102	1.02	1.06	0.99	2.42
	118	120	1.44	0.99	1.29	2.31
	120	103	1.87	0.86	0.34	2.13
	122	120	1.44	1.00	1.38	2.30
	123	161	1.73	0.87	1.12	1.97
	130	136	1.71	0.93	1.20	1.96
	133	105	1.21	1.10	1.63	2.82
	136	132	1.16	1.02	1.52	2.79
	139	112	0.53	0.97	2.53	6.58
	142	170	1.20	1.03	1.48	2.61
